# Gene Location, Expression, and Function of FNDC5 in Meishan Pigs

**DOI:** 10.1038/s41598-017-08406-y

**Published:** 2017-08-11

**Authors:** Chunbo Cai, Gaojun Xiao, Lili Qian, Shengwang Jiang, Biao Li, Shanshan Xie, Ting Gao, Xiaorong An, Wentao Cui, Kui Li

**Affiliations:** 1 State Key Laboratory of Agro Biotechnology, China Agricultural University, Beijing, 100193 P. R. China; 2 Institute of Animal Sciences, Chinese Academy of Agricultural Sciences, Beijing, 100193 P. R. China

## Abstract

Irisin is a new muscular regulatory factor that is generated by the cleavage of its precursor protein fibronectin type III domain-containing protein 5 (FNDC5). Irisin promotes fat consumption due to its stimulatory role in the browning of the adipocytes in mice. Currently, there is no report on FNDC5 functions in pigs as model animals. In this study, we investigated the expression patterns and functions of FNDC5 in Meishan pigs. Our results showed that FNDC5 gene in Meishan pigs contains five transcripts, all of which can be translated into functional intact irisin proteins. Porcine FNDC5 is mainly expressed in skeletal muscle, with the expression level being significantly higher during the embryonic and juvenile periods than in the adulthood stage. *In vitro* study showed that FNDC5 stimulates the proliferation and adipogenic differentiation of primary adipocytes isolated from Meishan pigs, and FNDC5 enhances the expression of browning marker genes during adipogenic differentiation. Our study was the first report on FNDC5 expression patterns and functions in pigs. Data from this study provide valuable information related to the study on FNDC5 functions and future development of novel treatment for obesity.

## Introduction

Obesity is a worldwide metabolic disorder that results in serious diseases such as type 2 diabetes^1^. The leading cause for obesity is due to overeating and/or less excises^2^, which then resulted in excess energy being stored in adipose tissue^3, 4^. Current studies show that adipose tissue can be classified into the following three types: white fat, brown fat, and beige fat^5^. The main function of white adipose tissue is to store excess energy, and is regarded as the main cause leading to obesity. The main function of brown fat is to provide the body with heat. Beige fat is a unique form of brown fat generated during the browning process of white fat, and it has similar function to brown fat^6, 7^. It is generally believed that the enhancement of browning in white fat can significantly result in consumption of the energy stored in white adipose tissue, and thus is a very effective approach to lose weight^8, 9^.

Skeletal muscle is recently being classified as a new endocrine organ, which secretes many cytokines called myokines that are involved in the regulation of body’s metabolic balance^10^. Initially, FNDC5 was believed to be involved in the regulation of skeletal muscle growth during embryonic development in mice^11^. Although FNDC5 is expressed primarily in skeletal muscle, it is also detected in other organs or tissues such as liver, fat, heart, and kidney^12, 13^. A recent publication^14^ reported that transgenic mice overexpressing PGC-1α in skeletal muscle can significantly increase FNDC5 expression and thus enhance the browning process of subcutaneous fat. It was also found that FNDC5 can be cleaved to form a novel protein, irisin, which increases the expression of the uncoupling protein 1 (UCP1) in adipose tissue and enhances the browning of white fats in mice^14^. Irisin protein contains 111 amino acids, and its amino acid sequence is highly conserved among a variety of species. FNDC5 is mainly synthesized in the skeletal muscle, and irisin generated by FNDC5 cleavage can be transferred into the adipose tissue through the circulatory blood system. The special effect of FNDC5 on adipose tissue makes it a unique therapeutic target to be studied and developed for treatment of obesity.

Exercise is a healthy and effective way to control and lose weight^15^. Many studies have demonstrated that exercise can increase the expression of FNDC5, which then enhances the synthesis and secretion of irisin in skeletal muscle^16, 17^. Irisin enters the adipose tissue through the blood system and enhances the browning process of white fat, resulting in the consumption of energy stored in fat and the subsequent reduction of fat deposition^18, 19^. However, many inconsistent results had been reported on the functional studies of serum irisin^20^. On one hand, some studies have reported a decrease in serum irisin levels in obese patients, and a negative correlation between irisin and body mass index (BMI)^21, 22^. On the other hand, there are reports indicating that serum irisin levels were positively associated with BMI^23, 24^. There were also a few studies on FNDC5′s function *in vitro*. Lee *et al*.^25^ reported that FNDC5 can increase the expression of browning genes in human primary adipocytes, and thus leads to an increase in the degree of browning process. Other studies^26, 27^ reported that irisin can increase the expression of UCP1 in rat primary adipocytes through P38 MAPK and ERK signaling pathways, leading to an enhanced browning of primary adipocytes in rats. However, current studies on FNDC5 had mainly focused on small model animals such as mice and rats, there were few studies reported in large animals^13, 28^.

Pigs are the closest species similar to humans^29^ and can be used as an ideal experimental animal model in the research and development of therapeutics to treat human diseases. Meishan pigs are a locally famous breed in China, and are well known for their high prolificacy and early sexual maturity, but the breed has a high percentage of carcass fat^30^. These unique qualities make Meishan pigs one of the best animal models to study obesity disease in humans. In this report, for the first time, we investigated the gene location, expression patterns of FNDC5, and its regulatory roles in the proliferation, differentiation, and the browning process of primary adipocytes in Meishan pigs.

## Results

### Identification of DNA sequence encoding the intact irisin protein in porcine genome

**Figure 1:**
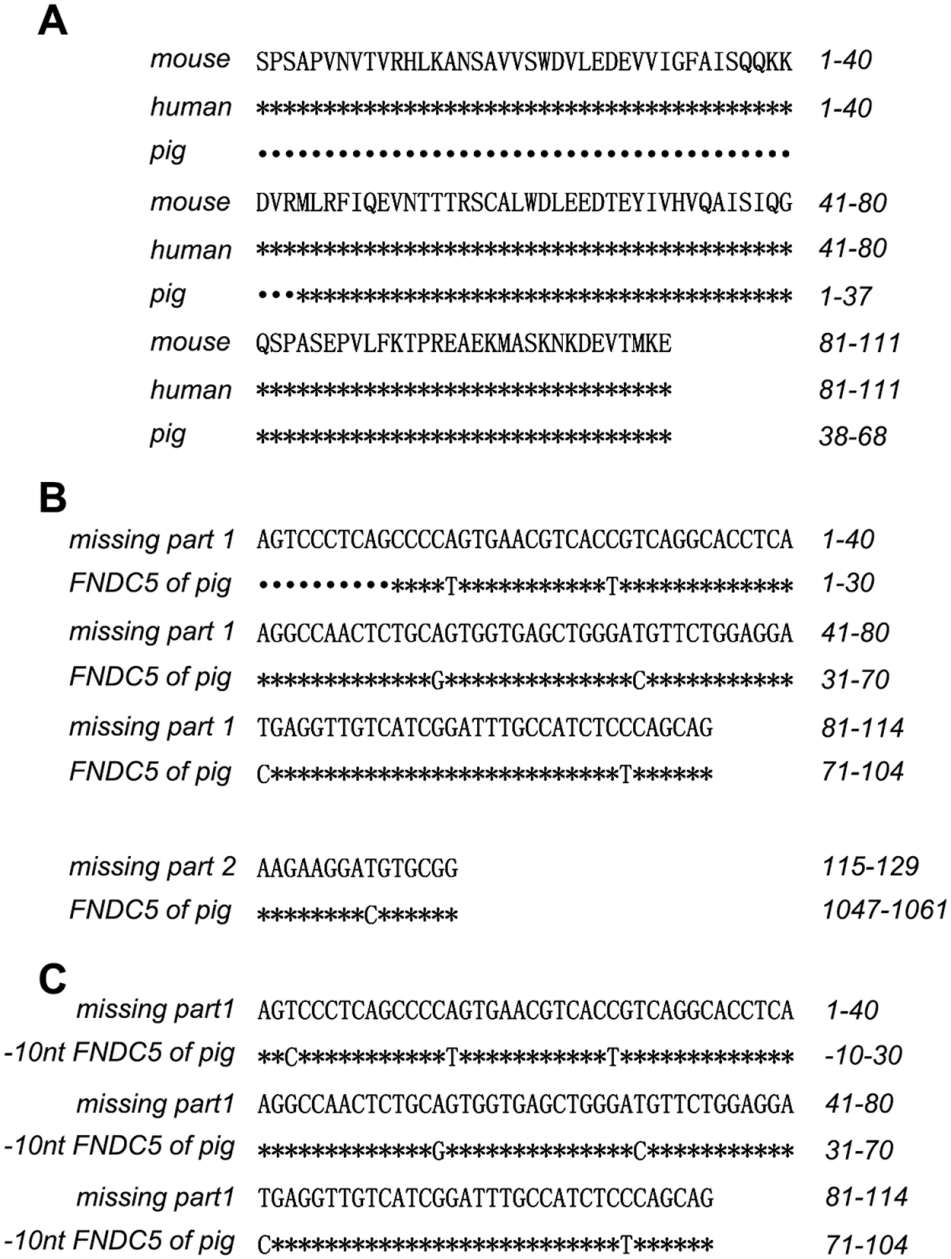
Identification of DNA sequences encoding intact irisin in porcine genome. (**A**) Amino acid sequence alignment of murine, human, and porcine irisin protein. *: indicating same amino acid; •: indicating missing amino acid. (**B**) DNA sequence encoding the missing 43 amino acids corresponding to the human genome compared to the NCBI database for porcine FNDC5. The missing sequence is divided into two parts, missing part 1 and missing part 2. *: indicating same base; •: indicating missing base. (**C**) Alignment of the missing part 1 DNA sequence encoding the missing 43 amino acids in human genome with DNA sequence of porcine FNDC5 from the NCBI database after adjustment of 10 bases up 5′ end. *: indicating same base; •: indicating missing base.

Amino acid sequences of irisin are highly conserved among species, with the homology of human and mouse being 100% (Fig. 1A). We have identified porcine FNDC5 gene sequence (see Gene ID: 100622587) and porcine FNDC5 protein sequence (see XP_005665248.1) on NCBI’s web site. By aligning porcine irisin amino acid sequence with those of human and mouse irisin, we noticed that the protein sequence of porcine irisin (XP_005665248.1) is incomplete, lacking the first 43 amino acids in the N-terminal (Fig. 1A). By looking at DNA sequence of human FNDC5 (Gene ID: 252995) on NCBI web site, we found that the corresponding gene sequence of human FNDC5 encoding the missing 43-amino acids identified in Meishan pigs is contained in two exons: missing parts 1 and 2 (Fig. 1B). These two partial deletions were then aligned with the DNA sequence of porcine FNDC5 on NCBI site (Gene ID: 100622587). The analysis result indicted that the DNA sequence of porcine FNDC5 on NCBI site lacks gene sequence for the synthesis of the complete irisin protein and lacks additional 10 base pairs (Fig. 1B). We then moved the DNA sequence of porcine FNDC5 on NCBI site to a 10 base pairs up 5′ end and aligned with the missing part 1 sequence (Fig. 1C) of human FNDC5, a match was observed, indicating that there exists a porcine DNA sequence that encodes the intact irisin protein.

### Detection of five FNDC5 transcripts in Meishan pigs

**Figure 2:**
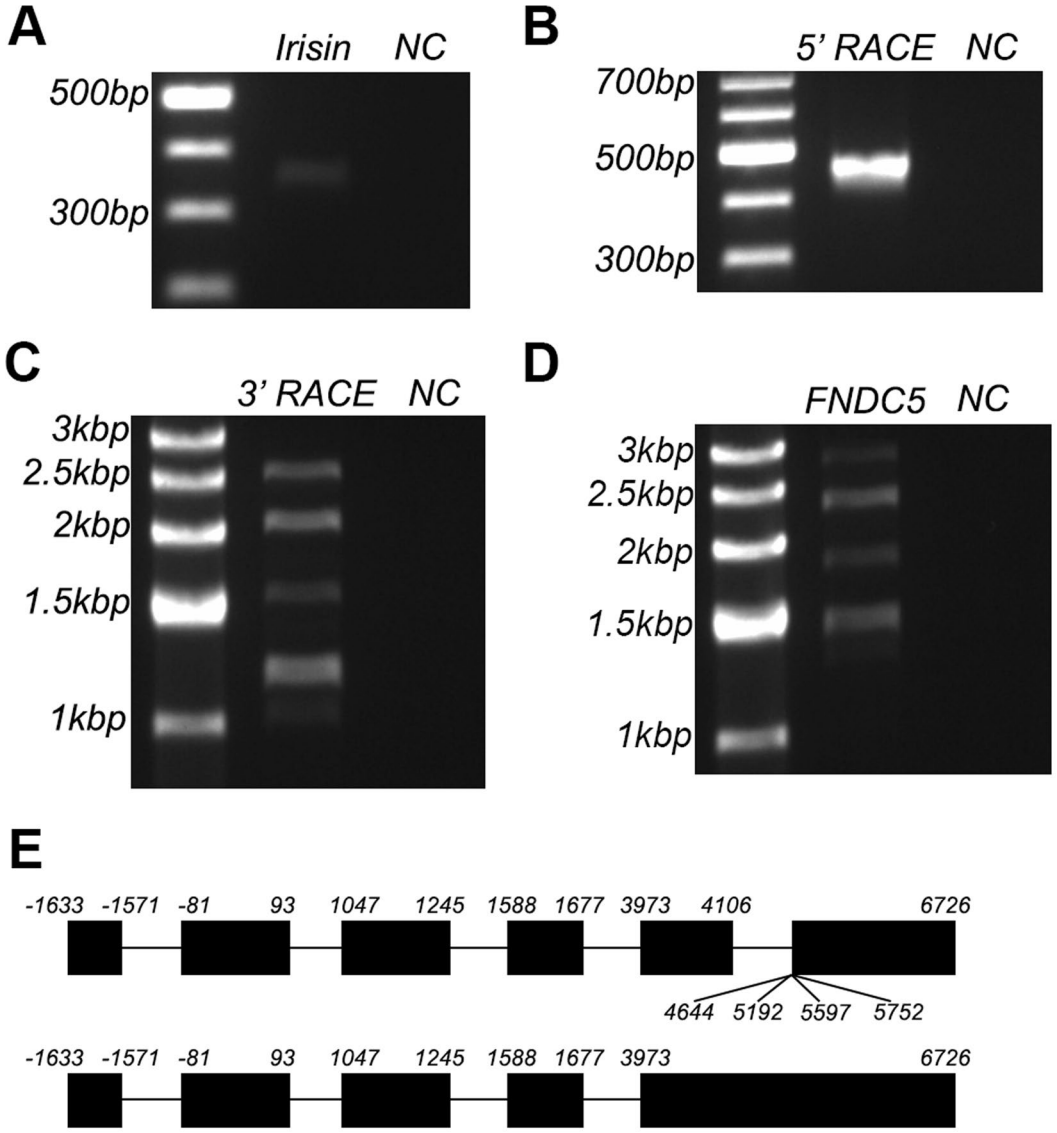
Detection of five FNDC5 transcripts in Meishan pigs. (**A**) RT-PCR results of irisin sequence in Meishan pigs, NC represents negative control group with no template. (**B**) 5′ RACE nested PCR results of FNDC5 from Meishan pigs, NC represents negative control group with no template. (**C**) 3′ RACE nested PCR results of FNDC5 from Meishan pig, NC represents negative control group with no template. (**D**) RT-PCR results from the full-length FNDC5 in Meishan pigs, NC represents negative control group with no template. (**E**) Different splicing patterns and locations of FNDC5 gene in Meishan pigs. Black bars: exons, black lines: introns. Numbers represent cleavage sites.

Based on the above sequence analysis, there exists a genomic DNA sequence encoding the complete porcine irisin protein. However, we do not know if the DNA sequence can be transcribed into any porcine irisin mRNA sequences. Thus, we designed primers (Irisin-F, Irisin-R) based on the information of the complete irisin DNA sequence (333 nucleotides) published on the NCBI site and performed PCR amplification using skeletal muscle cDNA from Meishan pigs as a template (Fig. 2A). Sequence results of PCR products showed that mRNA sequence for intact irisin protein can be transcribed in porcine skeletal muscle. To obtain the complete sequence of porcine irisin protein precursor FNDC5 mRNA, we then performed 5′ RACE, 3′ RACE, and nested PCR experiments, respectively. Results indicate that the 5′ end of the FNDC5 mRNA has only one splicing pattern (Fig. 2B), while the 3′ end has five different splicing patterns (Fig. 2C). If the DNA sequence of porcine FNDC5 published on NCBI site was moved to front (5′) end by 1633 bases, and then aligned with our RACE sequencing results (Fig. 2E), five transcripts are identified for porcine FNDC5 gene. The first 4 transcripts contained 6 exons, with exon 6 having 4 different splicing patterns. On the other hand, the fifth transcript contains only five exons. We then designed forward and reverse primers in the two ends of porcine FNDC5 (FNDC5-F, FNDC5-R) and performed PCR amplification using skeletal muscle cDNA as a template. Five PCR bands (Fig. 2D) were generated, confirming that there are indeed 5 transcripts for FNDC5 gene in Meishan pigs.

### Expression of porcine FNDC5 in skeletal muscle

**Figure 3:**
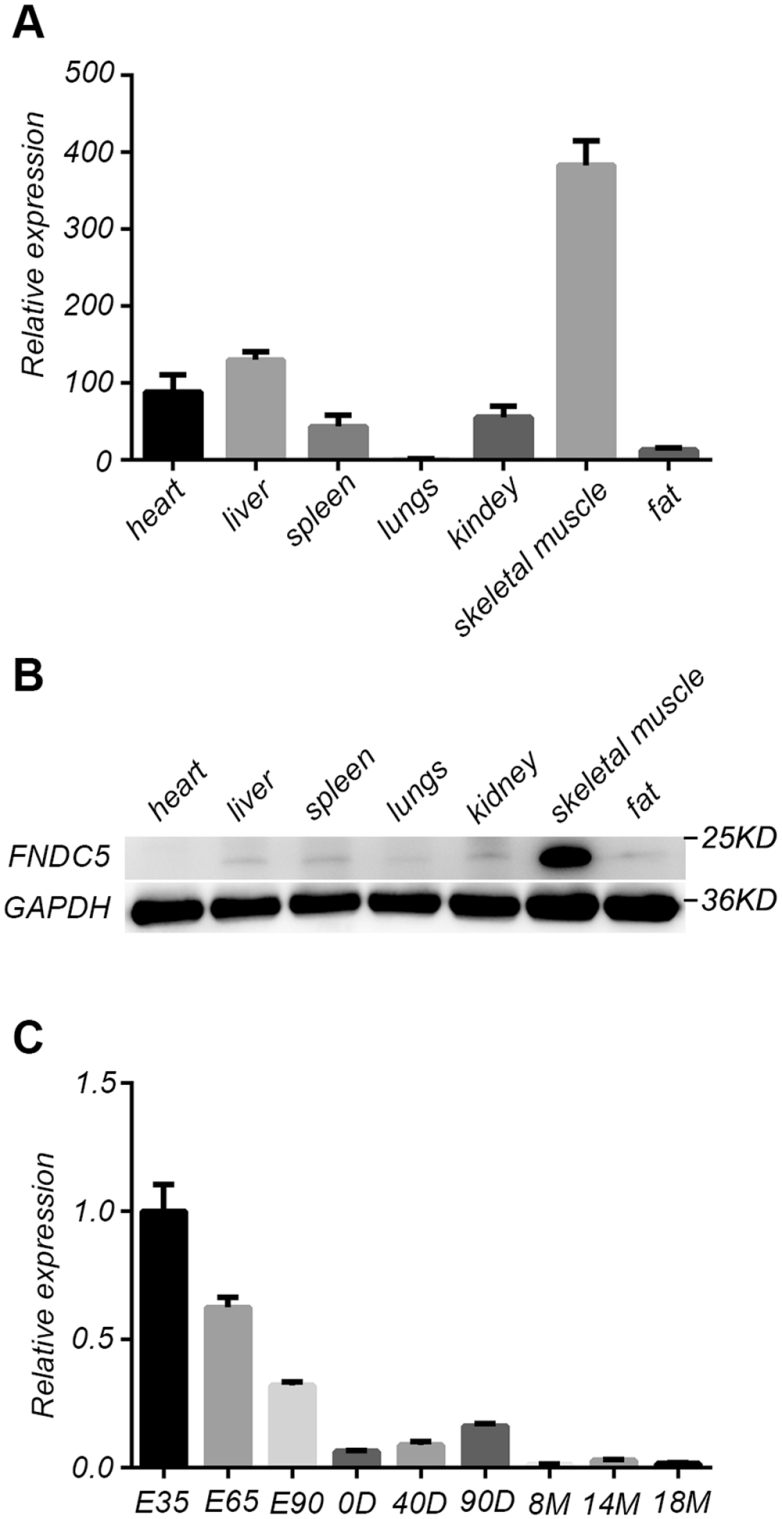
Different FNDC5 expression levels in organs and tissues from Meishan pigs. (**A**) Expression levels of FNDC5 in different tissues of Meishan pigs. Data are reported as mean ± SEM (n = 4). (**B**) Western blot results of FNDC5 in different tissues of Meishan pigs. (**C**) Expression levels of FNDC5 in skeletal muscle at different development stages of Meishan pigs. Data are reported as mean ± SEM (n = 4). E: embryo stage; D: days after birth; M: month after birth.

The expression of FNDC5 in different tissues from Meishan pigs was examined by qPCR. Results showed that FNDC5 was mainly expressed in skeletal muscle, with very low expression levels in heart, liver, and adipose tissue (Fig. 3A). The same result was obtained by analysis of Western blotting (Fig. 3B). We then also examined the expression of FNDC5 in skeletal muscle at different development stages (Fig. 3C). The expression level of FNDC5 in embryonic and juvenile periods was significantly higher than that in the adulthood.

### FNDC5 stimulates the proliferation of primary adipocytes isolated from Meishan pigs

**Figure 4:**
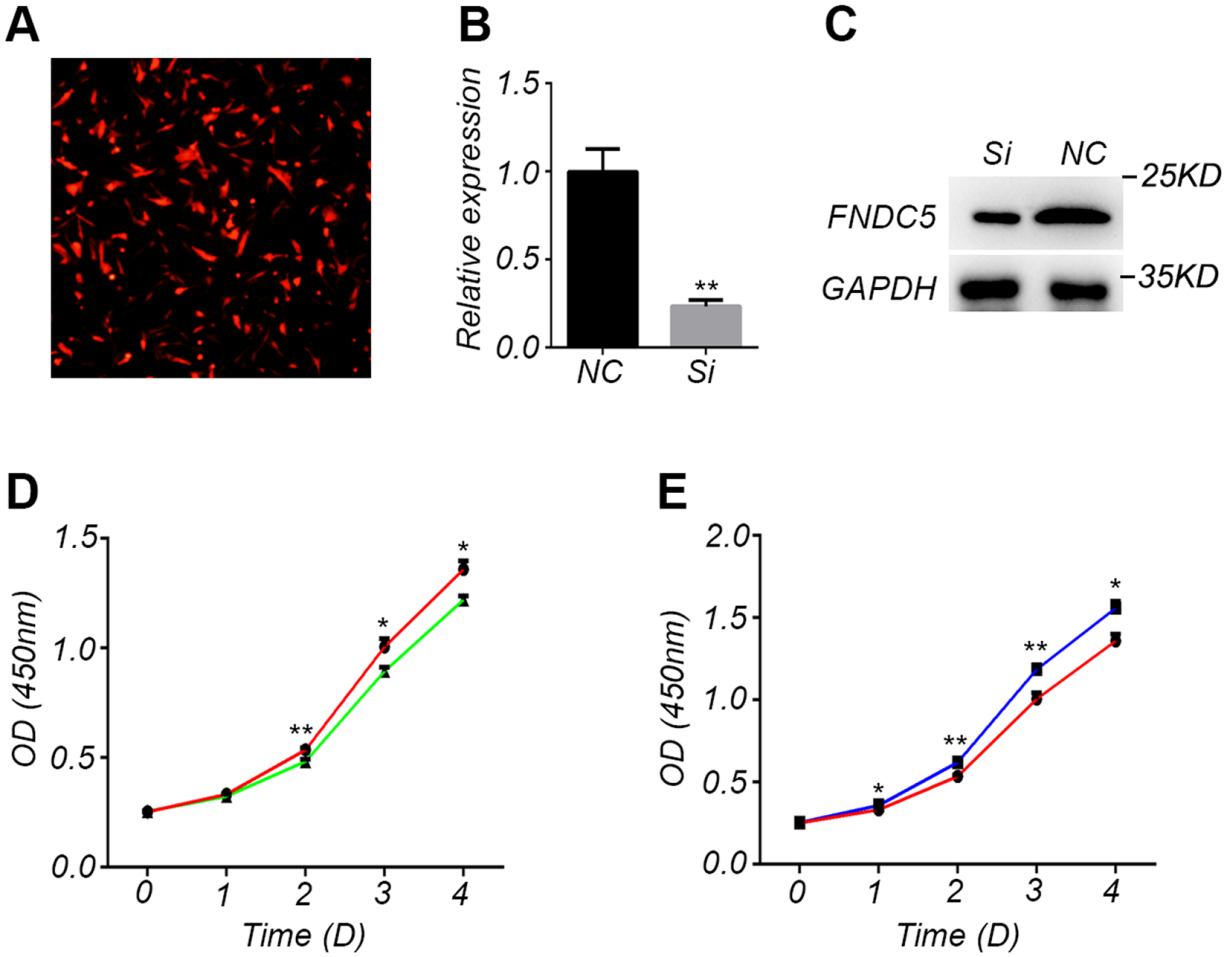
*In vitro* stimulation of proliferation of primary adipocytes by FNDC5 in Meishan pigs. (**A**) Lentiviral vector transfection efficiency. Red fluorescence represents cells stably transfected with FNDC5 interfering RNA. (**B**) FNDC5 expression levels as detected by qPCR in the control (NC) group and FNDC5 RNAi (Si) group in primary adipocytes of Meishan pigs. Data are reported as mean ± SEM (n = 3). *P < 0.05; **P < 0.01; ***P < 0.001. (**C**) Western blot results of FNDC5 in control (NC) group and RNAi (Si) group of primary adipocytes from Meishan pigs. (**D**) CCK8 assay was used to measure the proliferation of primary adipocytes in control (red line) group and RNAi (green line) group. Data are reported as mean ± SEM (n = 4), *P < 0.05; **P < 0.01. (**E**) CCK8 assay was used to measure the proliferation of primary adipocytes in the control (red line) group and FNDC5-treared (blue line) group. Data are reported as mean ± SEM (n = 4). *P < 0.05, **P < 0.01.

Currently, the function of FNDC5 in adipocytes is unclear. Thus, the effect of FNDC5 on the proliferation of primary adipocytes isolated from Meishan pigs was examined *in vitro*. The primary adipocytes were isolated from Meishan pigs and the effect of FNDC5 on proliferation was examined by either a direct inhibition (interfering RNA) method or by addition of recombinant FNDC5 (200 ng/ml). The lentiviral transfection efficiency was first performed using primary adipocytes and shown very high (Fig. 4A). Then the interference efficiency of the FNDC5 interfering RNA was examined on primary adipocytes. The results of qPCR and Western blot showed that the expression of FNDC5 was significantly inhibited in primary adipocytes transfected with lentivirus containing interfering RNA of FNDC5 (Fig. 4B,C). The proliferation of primary adipocytes was then determined by CCK-8 assay. Compared with the control group, the number of cells in the FNDC5 RNAi group started to decrease on day 2 and continued to decrease significantly on days 3 and 4 (Fig. 4D). On the other hand, the number of adipocytes in the culture medium supplemented with recombinant FNDC5 protein (200 ng/ml) started to increase significantly on day 1, and kept increasing more significantly on days 2, 3 and 4 (Fig. 4E). These *in vitro* results clearly indicate that FNDC5 can stimulate the proliferation of primary adipocytes isolated from Meishan pigs.

### FNDC5 stimulates adipogenic differentiation of primary adipocytes isolated from Meishan pigs

**Figure 5:**
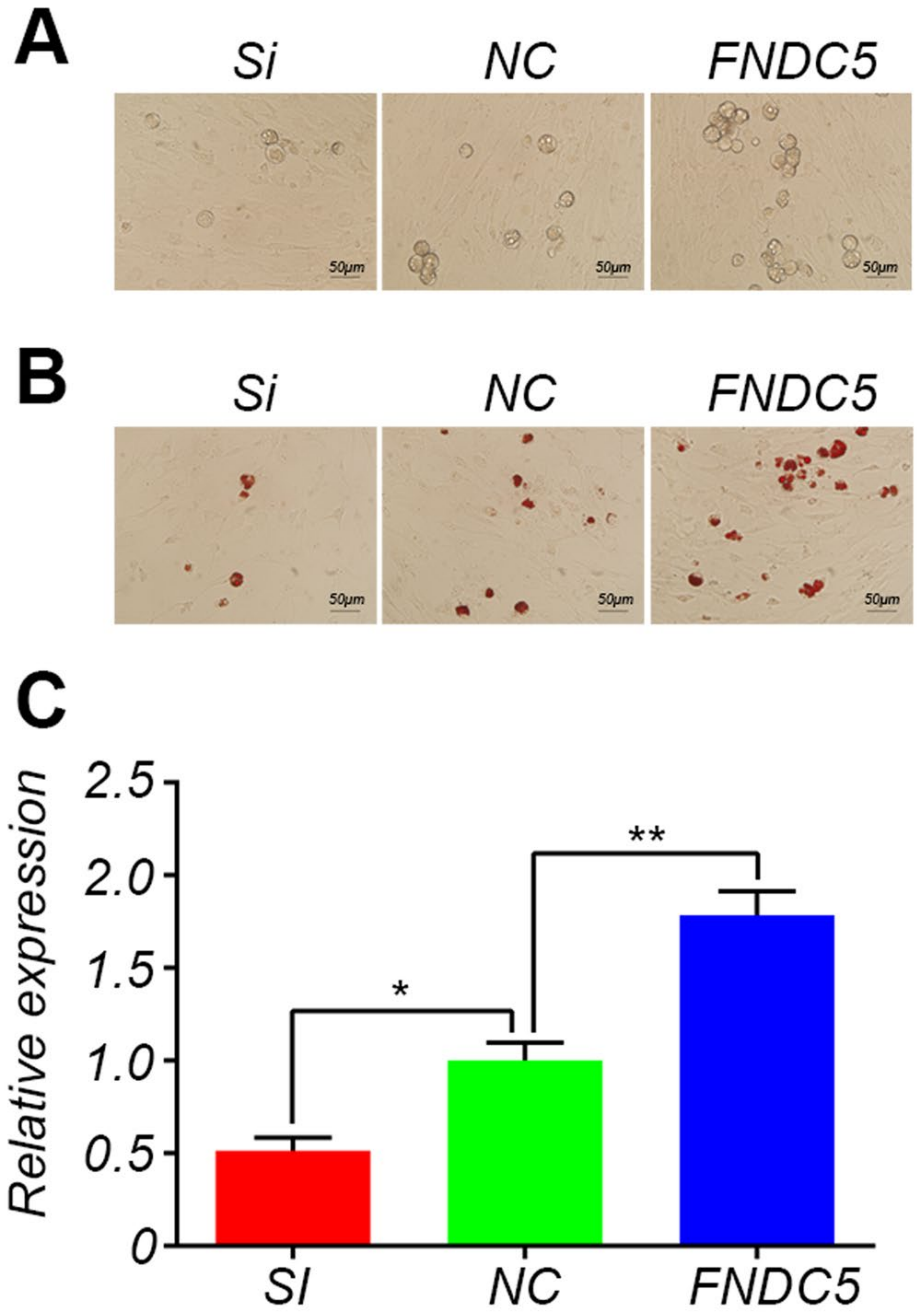
*In vitro* stimulation of adipogenic differentiation by FNDC5 in porcine primary adipocytes. (**A**) Photos showing Meishan porcine adipocytes following adipogenic differentiation on day 8. (**B**) Photos showing Meishan porcine adipocytes stained with oil red O following adipogenic differentiation on day 8. (**C**) The qPCR result of PPARγ. Data are reported as mean ± SEM (n = 3). *P < 0.05, **P < 0.01. Si: RNAi transfected group; NC: control plasmid transfected group; FNDC5: control plasmid transfected group supplemented with recombinant FNDC5 protein.

We also examined the effect of FNDC5 on adipogenic differentiation using primary adipocytes isolated from Meishan pigs. Primary adipocytes were transfected with RNAi and cultured in adipogenic differentiation medium for 8 days to induce adipogenic differentiation, followed by staining with oil red O. The number of lipid droplets was significantly reduced following the inhibition of FNDC5 by RNAi method (Fig. 5A,B). On the other hand, the addition of recombinant FNDC5 protein (200 ng/ml) to adipogenic differentiation medium resulted in a significant increase in the number of lipid droplets (Fig. 5A,B). Further, FNDC5 increased the expression of PPARγ (Fig. 5C).These *in vitro* results clearly indicate that FNDC5 can stimulate adipogenic differentiation of primary adipocytes in Meishan pigs.

### FNDC5 enhances the expression of browning marker genes of primary adipocytes isolated from Meishan pigs

**Figure 6:**
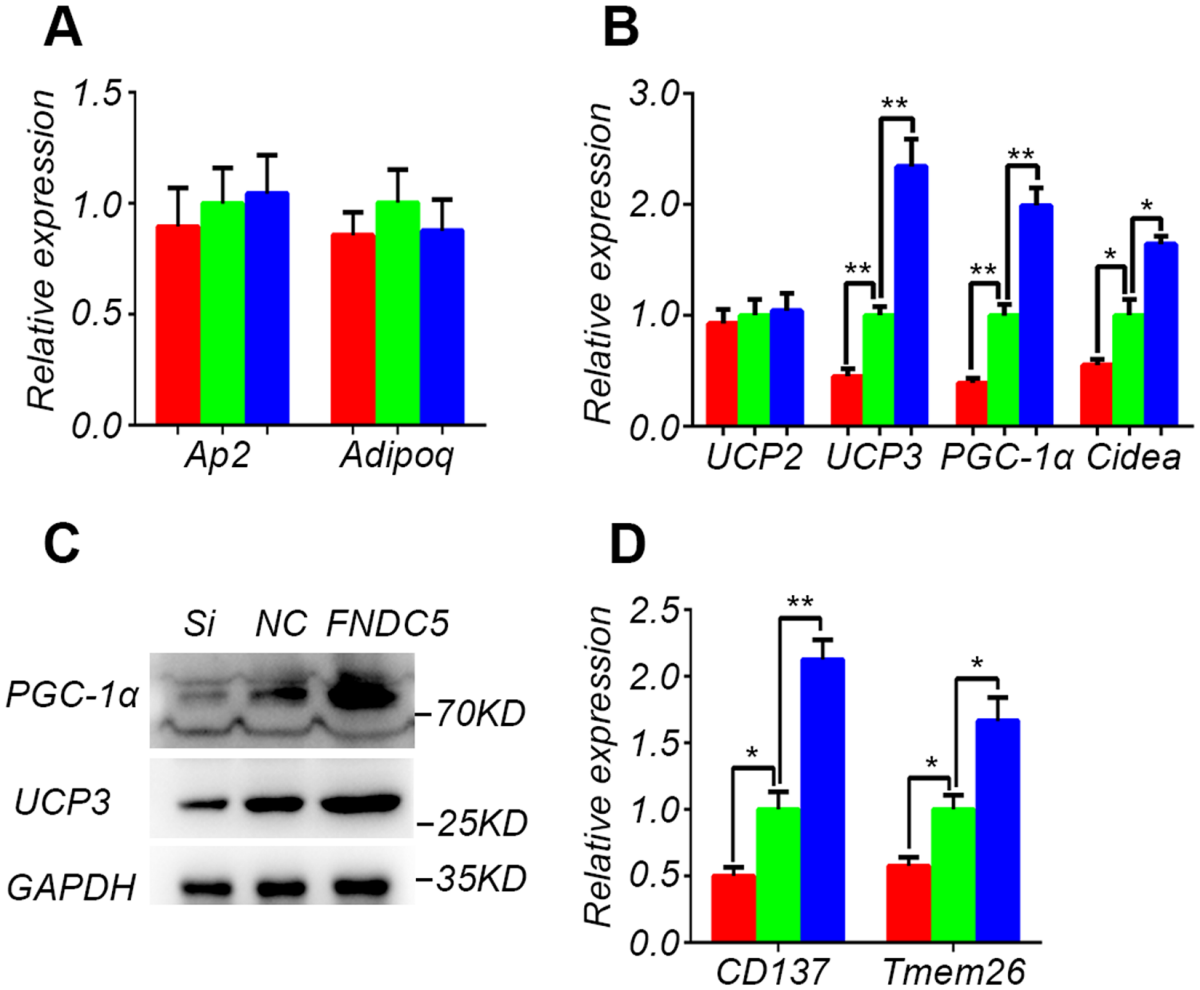
Enhancement of the browning of primary adipocytes by FNDC5 in Meishan pigs. Panels (A,B and D) represent the expression levels of marker genes for white fat, brown fat, and beige fat, respectively. Red column: RNAi transfected group; green column: control plasmid transfected group; blue column: control plasmid transfected group supplemented with recombinant FNDC5 protein. Data are reported as mean ± SEM (n = 3). *P < 0.05, **P < 0.01. (**C**) Western blot of browning marker gene products. Si: RNAi transfected group; NC: control plasmid transfected group; FNDC5: control plasmid transfected group supplemented with recombinant FNDC5 protein.

Fat is divided into white, brown, and beige fat based on appearance. We further examine the role of FNDC5 in adipogenic differentiation of primary adipocytes. The results from qPCR analysis showed that there was no significant change in the expression of white fat marker genes AP2 and Adipoq (Fig. 6A), while the expression levels of browning marker genes UCP3, PCG-1α and Cidea increased significantly (Fig. 6B). Western blot results also confirmed that the levels of brown fat marker proteins PGC-1α and UCP3 were significantly increased (Fig. 6C). Furthermore, the expression of beige fat marker genes CD137 and Tmem26 increased significantly (Fig. 6D). Therefore, our results indicate that FNDC5 can enhance the expression of browning marker genes of primary adipocytes in Meishan pigs.

## Discussions

Human diseases are complicated and diversified. Selection of appropriate animal as an experimental model to study a specific target disease is pivotal for understanding and development of new therapeutics to prevent and treat human diseases. Currently, the vast majority of scientific research focuses on mouse as an experimental model due to its short life cycle and easy experimental manipulation. However, there are shortages using mouse as an animal model since there are significantly obvious differences between human and mice. Recent studies have shown that pigs are not only similar to human in physiological structures^31, 32^, but also have a very high genetic similarity^29^ to humans. Additionally, pigs have much higher birth rates. These advantages make pigs an ideal experimental animal model to study human diseases. Most importantly, pigs have advantages in the research area of obesity. Like human, pigs eat all kinds of food including vegetables and meat. The sizes of fat cells as well as the fat distribution are very similar between pig and human. Both pig and human contain subcutaneous fat and visceral fat. Pig and human also have similar metabolism system and cardiovascular system. Therefore, use of pigs as an animal model is very useful in studying human obesity, although there is a shortcoming. UCP1 is a brown fat marker gene, it can consume the stored energy in the form of heat. However, porcine *UCP1* gene is disrupted and cannot form active UCP1 protein^33^. Thus, the presence of brown fats in pigs is still a controversial issue^34^. UCP2 and UCP3 are also members of the uncoupling protein family, and they can also stimulate fat consumption^35, 36^. J. Jia *et al*.^37^ reported that feeding pigs with a low protein diet leads to an increase in thermogenesis and a significant increase in expression of UCP2 and UCP3 in skeletal muscle and fat tissue. *In vivo*, ATP synthesis increased significantly in primary skeletal muscle cells isolated from UCP3 knockout mice without any increase in TCA cycle flux rate, implying an increased degree of mitochondrial energy coupling^38^. All these observations indicate that UCP3 can generate heat by enhancing energy consumption. Therefore, porcine UCP3 may replace UCP1’s role in generating heat. Beige adipocytes are a distinct type of thermogenic fat cell^39^. Beige adipocytes have their own marker genes such as CD137 and Tmem26^39^. Beige fat has the same function as brown fat and thus can consume fat to generate heat^39^. A previous study has shown that brown fat identified in human neck is beige fat^39^. Although pigs don’t contain intact UCP1 gene, their browning marker genes are all intact. Thus, pigs do not need UCP1 and may use other regulatory mechanisms to regulate beige fat to generate heat. In summary, although the use of pigs as an animal model to study obesity has its disadvantage, pigs are still much valuable than mice in the field of human obesity study.

FNDC5 gene sequence varies greatly among different species. From the NCBI database, we found that murine FNDC5 gene (Gene ID: 384061) contains 7673 bases and can be transcribed to produce two transcripts (ID: XM_006503212.3 and NM_027402.3), which are translated into two proteins with different amino acid sequences (XP_006503275.1 and NP_081678.1). Both murine FNDC5 proteins can be cleaved to generate an intact irisin protein containing 111 amino acids. Human FNDC5 gene (Gene ID; 252995) contains 10225 bases and can be transcribed into three transcripts (ID: NM_001171941.2, NM_001171940.1, and NM_153756.2) which are translated into three FNDC5 proteins with different amino acid sequences. The first two human FNDC5 proteins (ID: NP_001165411.2 and NP_715637.2) can be cleaved to generate an intact irisin protein containing 111 amino acids, but the third human FNDC5 protein (ID: NP_001165412.1) is cleaved to produce a non-intact irisin protein missing the first 43 amino acids at its N-terminal end. Porcine FNDC5 gene (ID: 100622587) contains 6726 bases identified from NCBI database, and this gene can generate two transcripts (ID: XM_005665191.2 and XM_005665192.2). However, neither of the two porcine mRNA sequences can be translated into intact irisin protein lacking 43 amino acids at its 5′ end, which is the same as the third human FNDC5 protein (ID: NP_001165412.1). RACE is an effective method to amplify full length mRNA using a known specific sequence at either the 3′ or the 5′-end of the mRNA^40^. We obtained five transcripts from porcine FNDC5 gene by performing RACE experiments. All these 5 transcripts contain the base sequence encoding an intact irisin protein, which is not consistent with the results predicted by the NCBI database. We further checked the Ensenble database and noted that there is one copy of FNDC5 transcript (ENSSSCT00000024523.1), which can be translated into a complete and an intact irisin protein (ENSSSCP00000021059). The sequence data from Uniprot database also shows a complete irisin protein (I3LLC7). Our results found that that there are five transcripts, each containing a different cleavage site in its non-codon region, and having no effect on synthesis of irisin protein. Therefore, we believe that the amino acid sequence of porcine irisin we identified in this study is accurate, while the deduced sequence from NBCI web site is not complete.

At present, treatment of obesity by enhancing the browning of white fats has a very attractive future^41^. Irisin^14^ and FNDC5^42–44^ are known to stimulate the browning of white fat, which increase the consumption of stored energy, and thus reduce fat mass. The cleavage of FNDC5 generates irisin containing 111 amino acid residues. Not many studies have been conducted on the effect of FNDC5 and irisin on cell proliferation and differentiation. Qiao *et al*.^27^ reported that irisin can stimulate rat primary osteoblast proliferation. Moon *et al*.^45^ reported that irisin can enhance the proliferation of murine hippocampal neuronal cells. These reports indicate that irisin stimulates cell proliferation. In this study, we observed that FNDC5 can stimulate the proliferation of primary adipocytes, indicating that FNDC5 and irisin have the same function in cell proliferation. Zhang *et al*.^26^ reported that irisin can enhance adipogenic differentiation of murine 3T3-L1 preadipocytes. However, Huh *et al*.^46^ reported a completely opposite result that irisin and FNDC5 inhibited human preadipocyte adipogenic differentiation. We found that porcine FNDC5 promoted *in vitro* adipogenic differentiation of primary adipocytes isolated from Meishan pigs. Therefore, it is a controversial issue regarding the regulatory roles of FNDC5 and irisin in adipogenic differentiation, and further investigation is required to clarify this issue in the future.

FNDC5 is mainly expressed in skeletal muscle, with very low expression level in adipose tissue. However, FNDC5 can promote the browning of white adipose tissue. On one hand, FNDC5 is cleaved to form irisin protein which is secreted into blood circulation and then enters the adipose tissues. Irisin promotes the browning of white fat brown and the consumption of fat. On the other hand, FNDC5/irisin play their biological roles through their own signaling pathways. There are very few reports on the FNDC5/irisin signaling pathways. Zhang *et al*.^26^ reported that irisin can promote browning of white adipocytes through mitogen-activated protein kinase p38 MAP kinase and ERK MAP kinase (MAPK) signaling. MAPK is a group of evolutionarily conserved serine/threonine protein kinases. Irisin plays its biological roles by activating MAPK and the subsequent signaling amplification. Liu *et al*.^47^ reported that irisin regulates glucose metabolism through PI3K/Akt signaling pathway. PI3K/Akt is a classical protein kinase signaling pathway that amplifies through the signal cascade and plays an important biological role. Therefore, although the expression level of FNDC5/irisin is low in adipocytes, their important biological functions can be accomplished through signaling pathways.

In summary, gene sequences, expression patterns, and functions of FNDC5 were studied in in Meishan pigs. Porcine FNDC5, which contains 5 transcripts, is mainly expressed in skeletal muscle, with very low expression levels in heart, liver, and fat. Additionally, the expression of FNDC5 in embryos and juvenile stages is significantly higher than that in adulthood. FNDC5 stimulates the proliferation and adipogenic differentiation of primary adipocytes in Meishan pigs, and enhances the browning of adipocytes. Our data on the expression patterns and functions of FNDC5 in Meishan pigs provide valuable information for further research and development of FNDC5 as a novel treatment of obesity.

## Materials and Methods

### Ethics statement

All pigs were fed with the same standard diet and raised under the same conditions. All experimental protocols related to animal work described in this study were reviewed and approved by the Institutional Animal Care and Use Committee (IACUC) at Institute of Animal Sciences, Chinese Academy of Agricultural Sciences. All experiments were performed in accordance with the approved guidelines for animal care and management of research projects.

### Reagents

Recombinant FNDC5 protein (Cat No: H00252995-P01) was from Abnova. The SMARTer RACE 5′/3′ Kit (Cat No: 634858) was purchased from Clontech Labs. Following antibodies were used in Western blot: primary antibodies against GAPGH (Cell Signal Technology, 2118), FNDC5 (Abcam, ab174833), UCP3 (Abcam, ab10985), PGC-1α (Santa, sc-13067), anti-mouse secondary antibody (Cell Signal Technology, 7076), and anti-rabbit secondary antibody (Cell Signal Technology, 7074). Cell Counting Kit (CCK-8, Cat No: CK04) was from Dojindo Molecular Technologies. Aadipogenic differentiation solution (Cat No: A10070-01) was from Gibco.

### RNA extraction and cDNA synthesis

Total RNA extraction was performed as previously described^48^. cDNA was synthesized using the tReverAid First Strand cDNA Synthesis Kit (K1622, Thermo) per the standard procedure.

### RT-PCR and qPCR

RT-PCR was performed per standard procedure. The primer sequences are shown in Table 1. qPCR was performed per the standard procedure using 7500 FAST Real-Time PCR System. Primer sequences are shown in Table 1. TPB^49^ was used as a porcine internal reference.

**Table 1:** Primer sequences used for RT-PCR and qPCR.

Primer Name	Primer sequence
Irisin-F	AGTCCCTCAGCCCCAGTGAA
Irisin-R	CTCCTTCATGGTCACCTCGT
Total FNDC5-F	ATGCACCCCGGGCCGCCC
Total FNDC5-R	TGCTCTAACTGGATCGGAGG
FNDC5-F	TGCAGGCCATCTCCATTCAG
FNDC5-R	ATATTGGCGGCAGAAGAGGG
UCP2-F	AGTGTGAGACCTGACGAAGC
UCP2-R	CCTTTCTCCCTGGATCTGC
UCP3-F	CAACAGGAAGTACAGCGGGA
UCP3-R	CACCATCTCGGCACAGTTCA
Cidea-F	GGGAGATAAGGGTCAGCGTG
Cidea-R	AAGCAGAGATGAAGAGGAAGCA
PGC-1α-F	CACCAGCCAACACTCAGCTA
PGC-1α-R	GAGGTGCACTTGTCTCTGCT
CD137-F	AAACAACCGTTTCTGAAGCCAG
CD137-R	TCAAGAGAGTCCCAGCACCT
AP2-F	GAAAGAAGTGGGAGTGGGCTT
AP2-R	GGTGGTTGTCTTTCCATCCCA
ADIPOQ-F	CGAGAAGCCTGGAGCACTAC
ADIPOQ-R	CCTTCAACCCCAGTCACTCC
PPARγ-F	GACCATTTCTGGGTCGCCT
PPARγ-R	AAAGTTGGTGGGCCAAAACG
TBP1-F	AACAGTTCAGTAGTTATGAGCCAGA
TBP1-R	AGATGTTCTCAAACGCTTCG

### 5′ and 3′ RACE and nested PCR

5′ and 3′ RACE were performed per the standard procedure provided by the SMARTer RACE 5′/3′ Kit. In the 5′ RACE experiment, a 5′ RACE adaptor sequence was added to the 5′-end of the cDNA. The adapter sequence matched with a universal primer UPS (CTAATACGACTCACTATAGGGC). PCR amplification was then carried out using the 5′ RACE specific primer GSP-R1 (CATGAACAGGACCACGACAATG) and the universal primer mixture UPM. Nested PCR was then performed by using 1:500 diluted PCR product as a template, the specific primer GSP-R2 (GCCCGTCCGCAGCTGTTGGTTCCTCC) and the universal primer UPS. Finally, the nested PCR product was ligated to the T vector and sequenced. 3′ RACE experiments were performed similarly per standard procedure provided by the Kit using 3′ RACE specific sequences GSP-F1(TGCAGGCCATCTCCATTCAG) and GSP-F2 (GAGGAACCAACAGCTGCGGACGGGC).

### Western blot

Protein extraction, sample preparation, and Western blot were performed per methods described previously^48^. Antibodies used in Western blot were described in the Reagents section above.

### Isolation of primary adipocytes

Newly born Meishan piglets were euthanized and soaked in 75% alcohol for 5 min. The scapula adipose tissue was separated aseptically, and cut into pieces, followed by adding 0.1% collagenase type I (GIBCO, 17100017) and incubation at 37 °C for 60 min. The digestion was terminated by adding fresh DMEM/F12 (Hyclone, SH30023.01B) medium containing 20% FBS (GIBCO, 10099-141) and 1% penicillin-streptomycin. Cells were then filtered (70 µm). The filtrate was centrifuged to remove supernatant, and the cell pellet was then resuspended in red blood cell lysis buffer. Centrifuge the cells after 5 min, discard supernatant, and resuspend pellet in DMEM/F12 medium containing 20% FBS and 1% penicillin-streptomycin. Cells were then transferred into T25 flasks, incubated at 37 °C and 5% CO_2_ and changed culture medium every 2 days.

### Lentiviral-mediated Transduction

The FNDC5 interfering RNA sequence (GCGATGCACAACTTTGCAAGT) was designed by GenePharma. Vector construction and lentiviral packaging were also performed by GenePharma. The primary adipocytes were inoculated in T25 flask. When cell confluency reached to 50%, DMEM/F12 medium containing 20% FBS was added along with 10 μL of virus stock and 5 μg/mL of Polybrene. Cells were then incubated at 37 °C and 5% CO_2_ for 24 hours. At 24 h hours and 48 hours, fresh DMEM/F12 medium containing 20% FBS was replaced, respectively. Then DMEM/F12 medium containing 20% FBS and 5 μg/mL of Puromycin was added. Cell culture was continued with fresh medium being changed every three days until stable expression of red fluorescent protein was observed.

### Cell viability assay

The cell viability assay was performed using the Cell Counting Kit-8 (CCK-8). The primary adipocytes from Meishan pigs were seeded in 96-well plates at a cell density of 1 × 10^4^ cells/cm^2^. Following incubation at 37 °C and 5% CO_2_ for 6 h to allow cells to become adherent, cells were then divided into 4 groups: day 0 group, day 1 group, day 2 group, and day 3 group. Fresh DMEM/F12 medium (100 µL/well) containing 20% FBS and CCK8 solution (10 µL/well) were directly added to wells of the day 0 group and incubated at 37 °C and 5% CO_2_ for 2 h. OD value at 450 nm was then measured. For day 1, 2 and 3 groups, cell culture was continued for 1, 2, and 3 days, respectively. Then fresh DMEM/F12 medium (100 µL/well) containing 20% FBS and CCK8 solution (10 µL/well) were added to wells of each group, and incubated at 37 °C and 5% CO_2_ for 2 h. OD value at 450 nm was then measured.

### Adipogenic differentiation and Oil Red O staining

The primary adipocytes from Meishan pigs were inoculated in 12-well plates at a cell density of 4 × 10^4^ cells/cm^2^ in fresh DMEM/F12 medium containing 20% FBS and 1% penicillin-streptomycin. When cells reached to 100% confluency, fresh adipogenic differentiation solution (A10070-01, Gibco) was added at 2 mL/well. Then change the fresh adipogenic differentiation solution every 3 days. When cells showed signs of clear fat droplets, adipogenic differentiation solution in each well was discarded, followed by wash with PBS. Each well was then added with 4% paraformaldehyde (2 mL/well) and fixed for 20 min. Following fixation, discard 4% paraformaldehyde, and then oil red O was added to stain for 10 min. Each well was then observed for the staining results under a microscope.

### Data statistics

All data are expressed as mean ± SEM and analyzed using unpaired 2-tailed Student’s t tests (P < 0.05).

## Electronic supplementary material

Supplementary information
