# Supplementary material for: Gene Location, Expression, and Function of FNDC5 in Meishan Pigs
Source: Sci Rep. 2017 Aug 11;7:7886. doi: 10.1038/s41598-017-08406-y (PMC5554266; doi:10.1038/s41598-017-08406-y)
Supplement: Supplementary file 1 — Supplementary information [file 41598_2017_8406_MOESM1_ESM.pdf]

Gene Location, Expression and Function of FNDC5 in Meishan Pigs

Chunbo Cai[1,2], Lili Qian[1,2] ,Shengwang Jiang[2], Gaojun Xiao[2], Biao Li[2], Shanshan Xie[2], Ting Gao[2], Xiaorong An[1*] , Wentao Cui[2*], Kui Li[2]

[1]State Key Laboratory of Agro Biotechnology, China Agricultural University, Beijing 100193, P. R. China. [2]Institute of Animal Sciences, Chinese Academy of Agricultural Sciences, Beijing 100193, P. R. China.

***Address for correspondence：**

Xiaorong An, Professor, State Key Laboratory of Agro Biotechnology, China Agricultural University. Beijing 100193, P. R. China. Tel: +86-010-62734661, E-Mail: xra@cau.edu.cn

Wentao Cui, associate professor, Institute of Animal Sciences, Chinese Academy of Agricultural Sciences, Beijing 100193, P. R. China. Tel: +86-010-62819480, E-Mail: cuiwentao@caas.cn

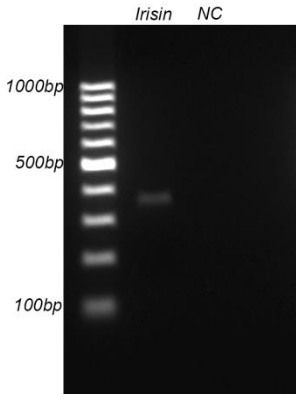

Supplement figure 1: Uncropped figure to Figure 2A in the main text, RT-PCR results of irisin sequence in Meishan pigs.

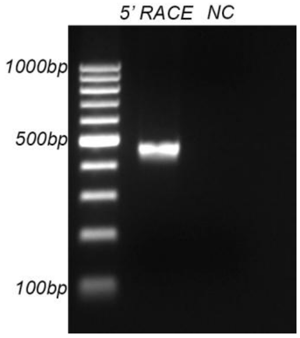

Supplement figure 2: Uncropped figure to Figure 2B in the main text, FNDC5 5' RACE nested PCR results from Meishan pigs.

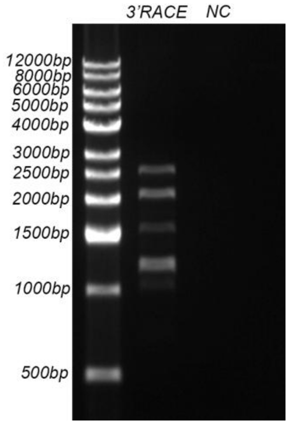

Supplement figure 3: Uncropped figure to Figure 2C in the main text, FNDC5 3' RACE nested PCR results from Meishan pig

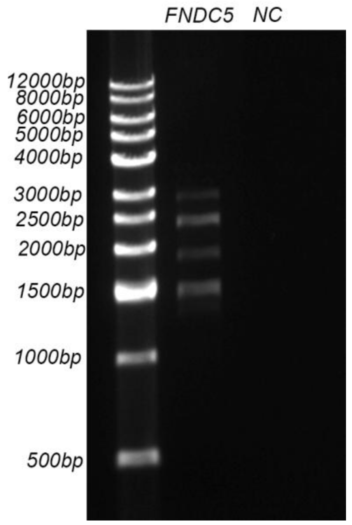

Supplement figure 4: Uncropped figure to Figure 2D in the main text, RT-PCR results from the full-length FNDC5 in Meishan pigs.

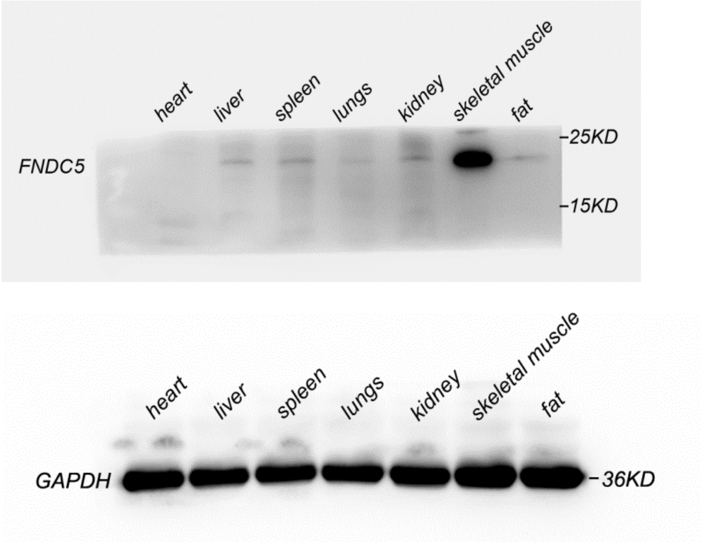

Supplement figure 5: Uncropped figure to Figure 3B in the main text, Western blot results of FNDC5 in different tissues of Meishan pigs

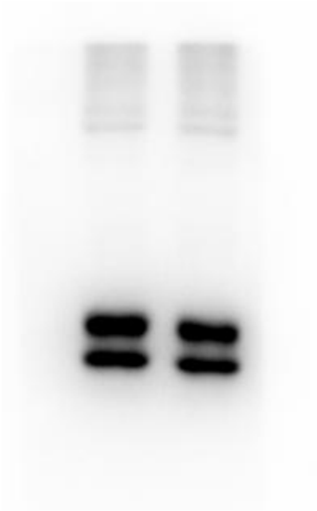

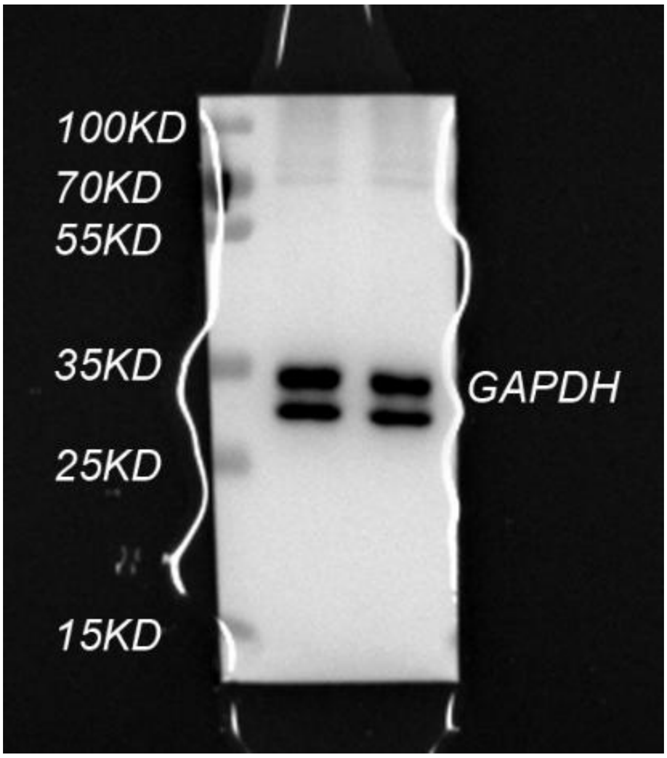
100KD
70KD
55KD
35KD
25KD
15KD
GAPDH

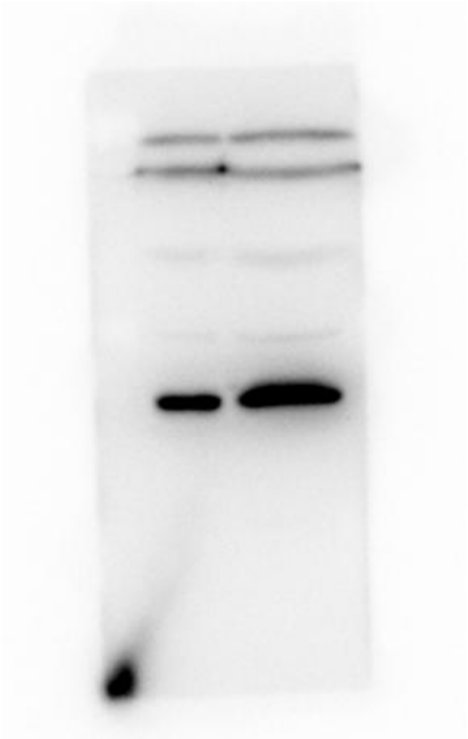

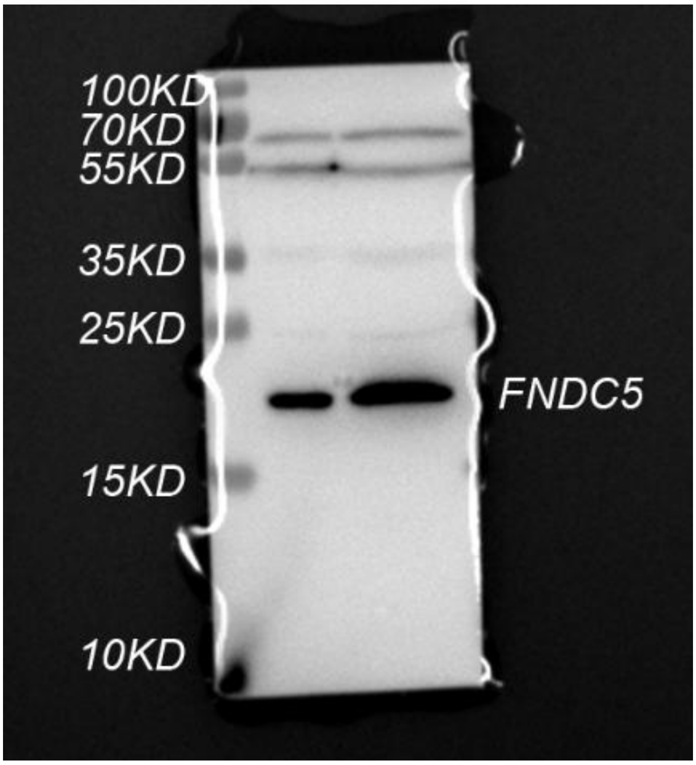
100KD
70KD
55KD
35KD
25KD
FNDC5
15KD
10KD

Supplement figure 6: Uncropped figure to Figure 4C in the main text, Western blot results of FNDC5 in control (NC) group and RNAi (Si) group of primary adipocytes from Meishan pigs.

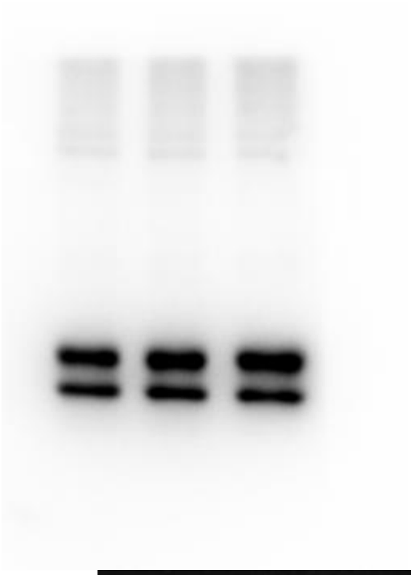

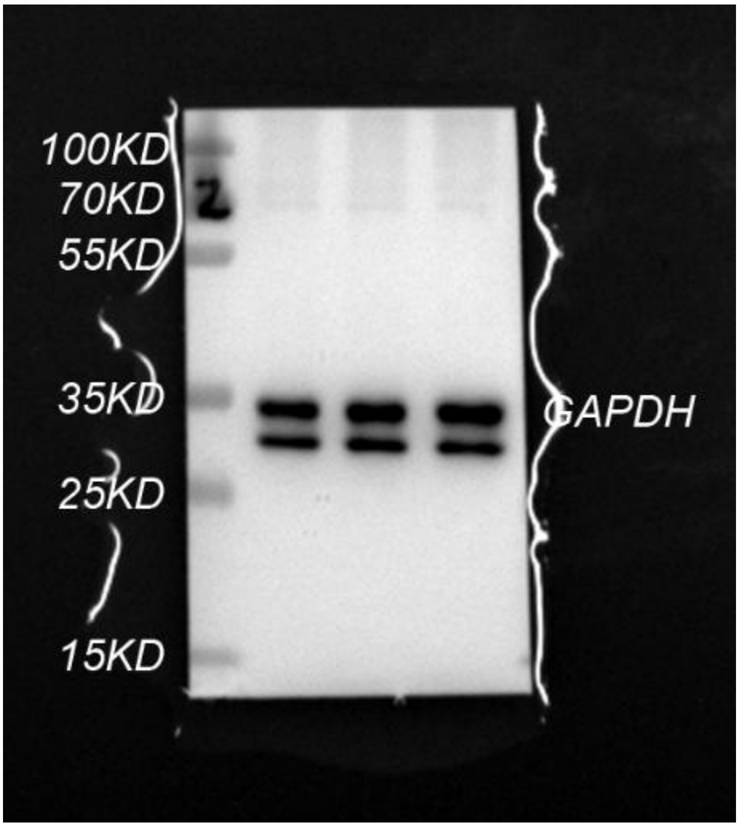

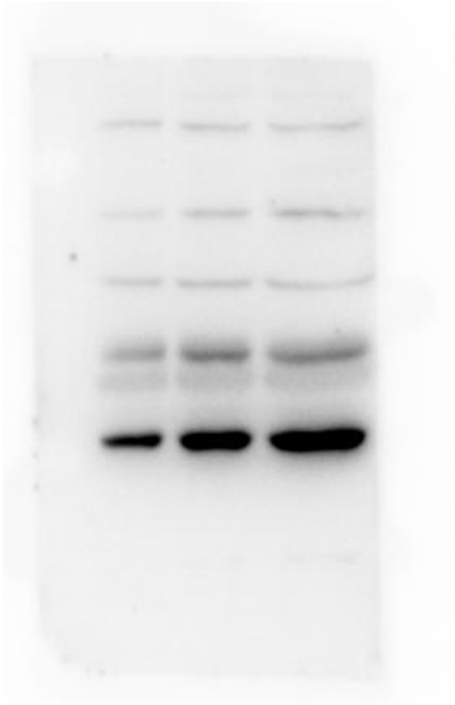

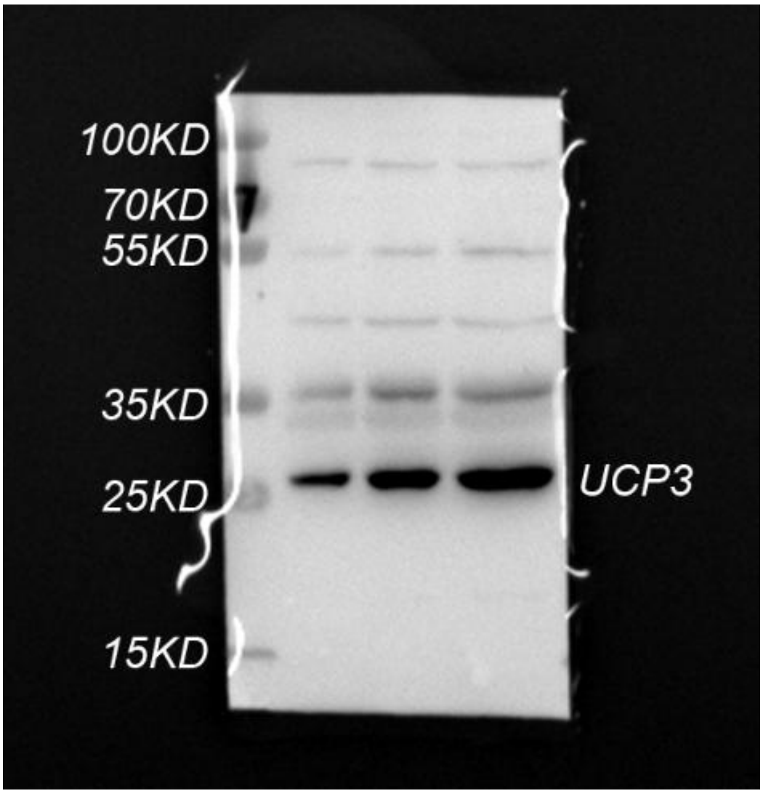
100KD
70KD
55KD
35KD
25KD
15KD
UCP3

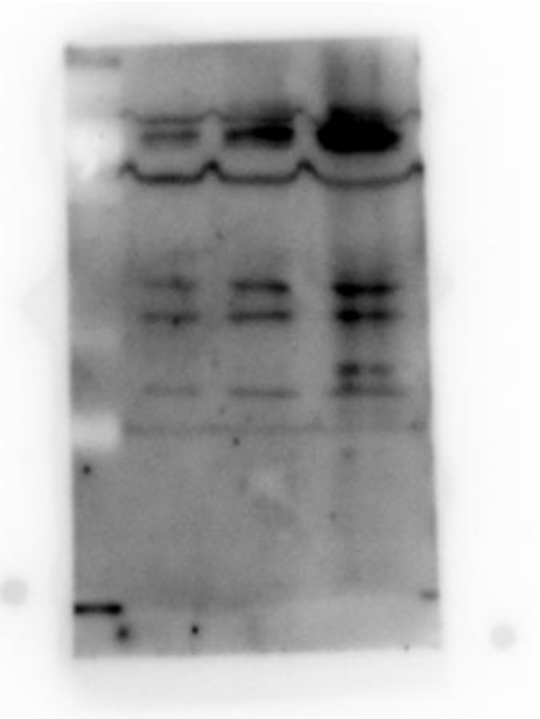

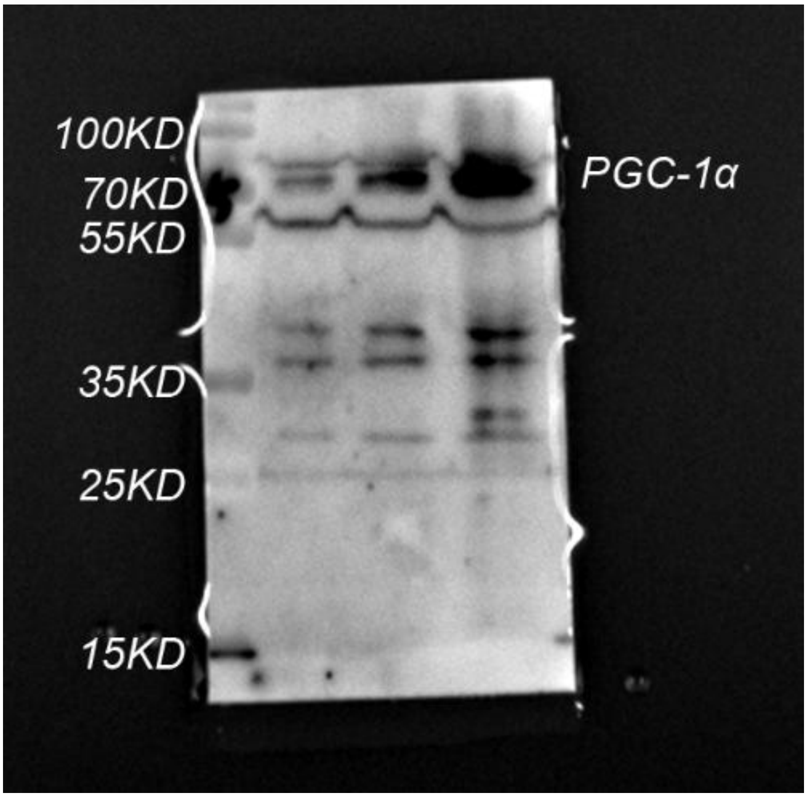

Supplement figure 7: Uncropped figure to Figure 6C in the main text, Western blot of browning marker gene products. Si: RNAi transfected group; NC: control plasmid transfected group; FNDC5: control plasmid transfected group supplemented with recombinant FNDC5 protein.
